# E-cadherin loss induces targetable autocrine activation of growth factor signalling in lobular breast cancer

**DOI:** 10.1038/s41598-018-33525-5

**Published:** 2018-10-18

**Authors:** Katy Teo, Laura Gómez-Cuadrado, Milou Tenhagen, Adam Byron, Max Rätze, Miranda van Amersfoort, Jojanneke Renes, Eric Strengman, Amit Mandoli, Abhishek A. Singh, Joost H. Martens, Hendrik G. Stunnenberg, Paul J. van Diest, Valerie G. Brunton, Patrick W. B. Derksen

**Affiliations:** 1 Cancer Research UK Edinburgh Centre, Institute of Genetics and Molecular Medicine, University of Edinburgh, Edinburgh, United Kingdom; 2 Department of Pathology, University Medical Center Utrecht, Utrecht, The Netherlands; 3 Department of Molecular Biology, Faculty of Science, Nijmegen Centre for Molecular Life Sciences, Radboud University, Nijmegen, The Netherlands

## Abstract

Despite the fact that loss of E-cadherin is causal to the development and progression of invasive lobular carcinoma (ILC), options to treat this major breast cancer subtype are limited if tumours develop resistance to anti-oestrogen treatment regimens. This study aimed to identify clinically targetable pathways that are aberrantly active downstream of E-cadherin loss in ILC. Using a combination of reverse-phase protein array (RPPA) analyses, mRNA sequencing, conditioned medium growth assays and CRISPR/Cas9-based knock-out experiments, we demonstrate that E-cadherin loss causes increased responsiveness to autocrine growth factor receptor (GFR)-dependent activation of phosphatidylinositol-4,5-bisphosphate 3-kinase (PI3K)/Akt signalling. Autocrine activation of GFR signalling and its downstream PI3K/Akt hub was independent of oncogenic mutations in *PIK3CA*, *AKT1* or *PTEN*. Analyses of human ILC samples confirmed growth factor production and pathway activity. Pharmacological inhibition of Akt using AZD5363 or MK2206 resulted in robust inhibition of cell growth and survival of ILC cells, and impeded tumour growth in a mouse ILC model. Because E-cadherin loss evokes hypersensitisation of PI3K/Akt activation independent of oncogenic mutations in this pathway, we propose clinical intervention of PI3K/Akt in ILC based on functional E-cadherin inactivation, irrespective of activating pathway mutations.

## Introduction

Invasive lobular carcinoma (ILC) is a major luminal breast cancer subtype accounting for approximately 15% of all breast cancers. Loss of E-cadherin expression is a hallmark of ILC that is already evident in lobular carcinoma *in situ* (LCIS), a lesion that is believed to precede ILC formation^1,2^. Conditional mouse models have demonstrated that E-cadherin loss is causal to the development and progression of lobular breast cancer. Subsequent studies using mouse and human ILC models have shown that tumour progression is in part due to anchorage independence triggered by p120-catenin-dependent activation of RhoA and Rock1^3,4^. Loss of E-cadherin expression is observed in the vast majority of lobular breast cancers, mostly due to inactivating *CDH1* mutations and subsequent loss of heterozygosity, or epigenetic silencing of the E-cadherin promoter^5^. As a result of E-cadherin inactivation, the adherens junction (AJ) is no longer functional, leading to disruption of epithelial integrity and acquisition of tumour-promoting events such as anchorage independence, angiogenesis and tumour cell invasion^6^.

Another major driver in breast cancer is the phosphatidylinositol-4,5-bisphosphate 3-kinase (PI3K) pathway, which can be activated through loss of phosphatase and tensin homolog (PTEN) function or activating mutations in PI3K subunits or their downstream effectors. ILCs represent a subgroup of tumours in which the mutation rate of *PIK3CA* (48%) and genomic loss of *PTEN* (13%) is higher than in matched IDCs (37% and 11%, respectively)^7^. In addition, although the underlying activation cue remains unknown, increased activation of PI3K signalling was linked to specific subtypes, including basal-type, HER2-positive and ILC tumours^7,8^. These findings have triggered an increase in clinical trials to target PI3K, Akt or mechanistic target of rapamycin (mTOR)^9–11^. Given the broad occurrence of PI3K/Akt pathway mutations, clinical intervention of this pathway has not been tailored for a specific breast cancer subtype. Also, despite the recent insight into the oncogenic pathways underpinning ILC, there is no targeted intervention strategy to treat ILC once tumours are refractory to hormone receptor antagonists. Although next-generation sequencing and mRNA expression profiling have provided a comprehensive and detailed genomic and transcriptional landscape of lobular and ductal breast cancers, they have yielded limited direct insight into pathway and protein activation. Moreover, while recent studies have coupled protein expression to patient survival^12,13^, they did not specifically report on ILC.

Here, we have studied human and mouse models of ILC to delineate the consequences of E-cadherin loss to the activation of druggable signalling pathways. We find that growth factor signals are hyperactivated upon E-cadherin loss, independent of somatic activating mutations in downstream effectors. Our study advocates clinical implementation of drugs targeting the PI3K/Akt axis in ILC, irrespective of oncogenic pathway mutations.

## Results

### Pathway analysis reveals activation of PI3K/Akt signalling in ILC cells

**Figure 1:**
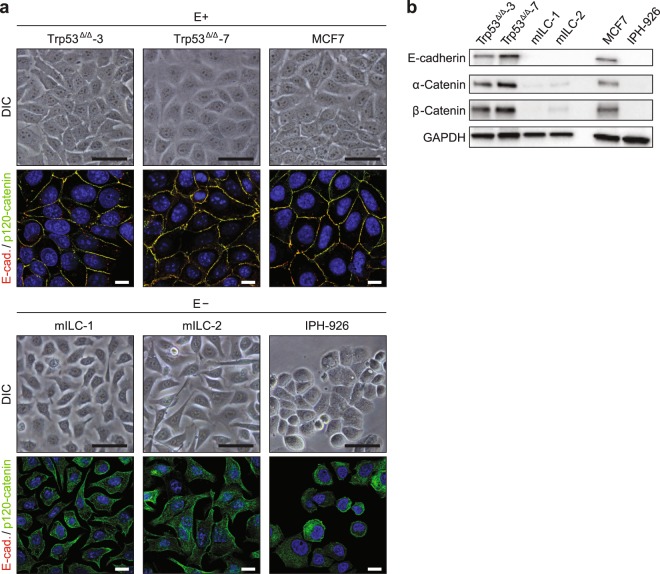
Breast cancer cells used in this study. (**a**) Differential interference contrast (DIC) microscopy images and merged immunofluorescence microscopy images for E-cadherin (E-cad.; red) and p120-catenin (green) expression in mouse (left and middle panels) and human (right panels) breast cancer cell lines. E-cadherin-expressing (E+; upper panels) and E-cadherin mutant (E−; lower panels) cells are grouped accordingly. Scale bars for DIC, 20 µm; scale bars for immunofluorescence, 10 µm. (**b**) Expression of the AJ components E-cadherin, α-catenin and β-catenin was assessed by western blotting. GAPDH served as a loading control.

To study the effect of E-cadherin loss on downstream pathway activation, we made use of well-characterised cell lines from metastatic mouse and human ILC and their non-metastatic E-cadherin-positive counterparts (Fig. 1). These included mouse ILC (mILC) lines that were derived from E-cadherin-deficient mammary tumours and cell lines derived from non-invasive tumours that developed in mammary-specific p53 conditional knock-out mice (Trp53^Δ/Δ^ cells)^14,15^. As a model of human ILC, we used IPH-926 cells^16^. MCF7 cells were used as a control, E-cadherin-expressing, non-metastatic human breast cancer cell line (Fig. 1).

**Figure 2:**
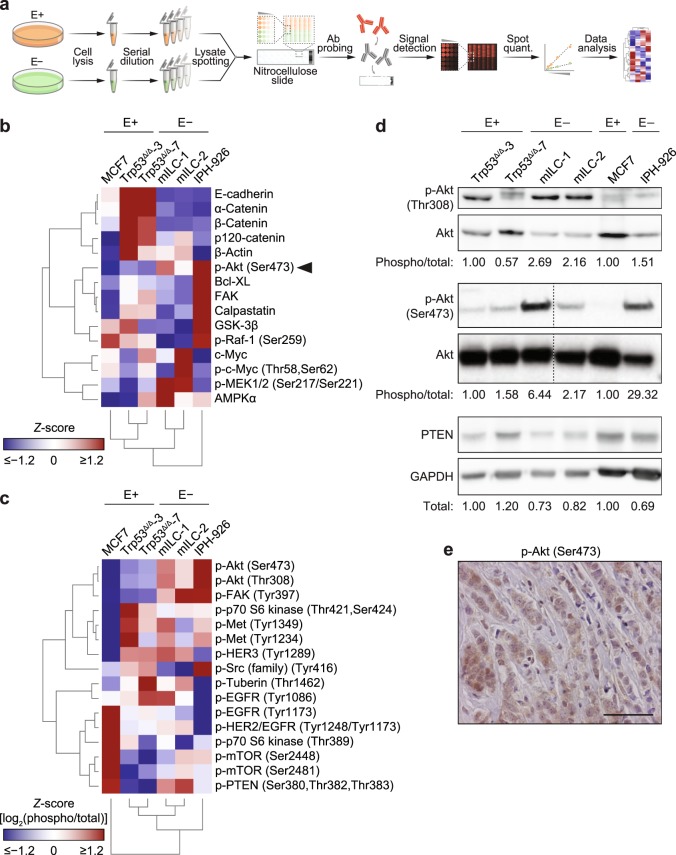
Differential protein expression and phosphorylation in the context of E-cadherin expression. (**a**) Experimental workflow for the RPPA analysis. After collection, dilution and spotting of the cell lysates, each of 16 sub-arrays (pads) per nitrocellulose slide were probed with a different validated primary antibody (Ab). A fluorescent secondary antibody was used for signal detection and quantification (quant.). Mean intensities of the biological replicates were used to perform cluster analysis. E+, E-cadherin-expressing cells; E−, E-cadherin-negative cells. (**b**) Hierarchically clustered heat map showing the relative levels of differentially regulated proteins and phosphoproteins (*Q* = 0.05) in whole cell lysates from mouse (Trp53^Δ/Δ^-3, Trp53^Δ/Δ^-7, mILC-1, mILC-2) and human (MCF7, IPH-926) cell lines as determined by RPPA. (**c**) Hierarchically clustered heat map showing the relative levels of phosphoproteins related to the Akt signalling pathway. Heat maps display the relative expression (*Z*-scores) of proteins or phosphoproteins (red, up-regulated; blue, down-regulated). (**d**) Western blot analysis of differentially regulated proteins and phosphoproteins identified by RPPA. Phosphorylation levels of Akt (p-Akt; Thr308 and Ser473) were assessed and normalised over the corresponding total protein levels, while PTEN expression levels were normalised over GAPDH levels. For mouse cells, normalised phosphoprotein levels in Trp53^Δ/Δ^-3 cells were set to 1; for human cells, normalised phosphoprotein levels in MCF7 cells were set to 1. For analysis of phospho-Akt (Ser473), blot lanes for additional mILC sub-clones were removed, as denoted by the dashed lines. (**e**) Representative immunohistochemistry image of expression of phospho-Akt (Ser473) in ILC patients (see Table 2). Scale bars, 50 µm.

To examine the effect of E-cadherin inactivation on protein expression, post-translational modifications and downstream pathway activation, we used reverse-phase protein array (RPPA) analysis to provide a relatively high-throughput antibody-based platform for the quantification of protein expression and phosphorylation status (Fig. 2a). Expression and phosphorylation of key signalling proteins were assayed using a panel of 120 antibodies directed against established oncogenic pathways such as growth factor receptor (GFR) signalling, stress response, cell adhesion and apoptosis (Supplementary Figs S1 and S2 and Supplementary Tables S1–S3). Unsupervised hierarchical cluster analysis of the significantly differentially regulated proteins and phosphoproteins identified a distinct separation of the E-cadherin-expressing cell lines and the E-cadherin mutant ILC cell lines (Fig. 2b). As reported previously^3^, we noted that expression levels of α-catenin, β-catenin and p120-catenin were decreased in E-cadherin mutant ILC cells (Fig. 2b), a finding that served as an internal control for the RPPA (see also Fig. 1b). E-cadherin-negative cells consistently showed higher activation (phosphorylation) of Akt (Fig. 2b–d), while expression of PTEN was lower in ILC cells when compared to E-cadherin-expressing breast cancer cells (Fig. 2d and Supplementary Table S2).

Finally, we analysed expression of the proteins that showed elevated expression in ILC cells using a tissue microarray (TMA) containing 129 primary ILC samples and 30 LCIS samples (Table 1). In agreement with the RPPA and western blotting data from the human and mouse cell line panel, we observed that levels of active Akt were common in LCIS and ILC (Fig. 2e and Table 2). Interestingly, PTEN expression was reduced or lost in the majority of ILC samples when compared to E-cadherin-expressing cells (Table 2), suggesting that inactivation of PTEN may be induced during ILC progression. In conclusion, using a comprehensive set of mouse and human cell lines and primary tumour samples, we have identified increased activation of a distinct set of oncogenic pathways and proteins in ILC.

**Table 1:** Clinicopathological features of the 129 ILC patients studied for phospho-Akt (Ser473) and PTEN expression.

Clinicopathological characteristic		Number	Proportion (%)
Age (years)	Mean	57	—
	Range	32–83	—
Histologic grade	1	12	9.3
	2	76	58.9
	3	35	27.1
Lymph node status	Negative	68	52.7
	Positive	57	44.2
Metastasis	Negative	86	66.7
	Positive	9	7.0
ER status (10% cutoff)	Negative	12	9.3
	Positive	107	82.9
PR status (10% cutoff)^a^	Negative	35	27.1
	Positive	83	64.3
HER2 (3+)	Negative	86	66.7
	Positive	7	5.4

^a^PR, progesterone receptor.

**Table 2:** Expression of phospho-Akt (Ser473) and PTEN in ILC and LCIS.

Antigen		ILC		LCIS		*P*
		Number	Proportion (%)	Number	Proportion (%)	
p-Akt (Ser473)	Negative	42	35	3	10	0.007
	Positive	75	65	26	90	
PTEN	Low/absent	61	76	12	40	<0.001
	Positive	19	24	18	60	

### PI3K/Akt pathway activation is a consequence of E-cadherin loss

**Figure 3:**
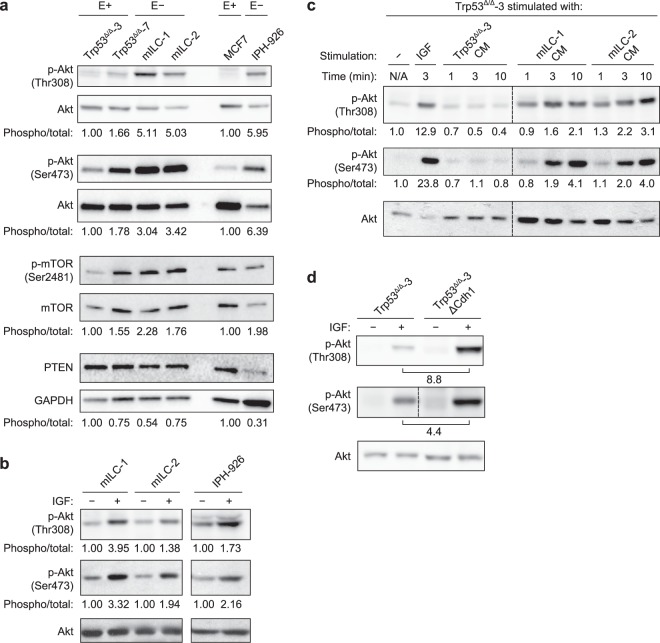
Autocrine growth factor-dependent PI3K/Akt activation is a direct consequence of E-cadherin loss in ILC. (**a**) PI3K/Akt signalling in E-cadherin-expressing (E+) and E-cadherin mutant (E−) ILC cells. Shown are the phosphorylation status of Akt (Thr308 and Ser473), the phosphorylation status of mTOR (Ser2481) and the expression of PTEN. Quantification of mouse protein expression is relative to the levels in Trp53^Δ/Δ^-3 cells and human protein expression is relative to the levels in MCF7 cells. Phosphorylation of Akt (Thr308 and Ser473) and mTOR (Ser2481) were normalised over total protein levels; PTEN levels were normalised over GAPDH levels. (**b**) Phosphorylation of Akt (Thr308 and Ser473) is induced upon IGF stimulation of E-cadherin-negative cells. Quantification of protein levels is relative to the levels in unstimulated cells. Separate gels were run with the mILC and IPH-926 cell lines. (**c**) Stimulation of Trp53^Δ/Δ^-3 cells with IGF and mILC-conditioned medium (CM) induces phosphorylation of Akt. Akt phosphorylation levels in unstimulated cells were set to 1. Replicate blot lanes for cells stimulated with conditioned medium were removed, as denoted by the dashed lines. N/A, not applicable. (**d**) E-cadherin knockout (ΔCdh1) in Trp53^Δ/Δ^-3 cells increases basal levels of phospho-Akt. Stimulation of serum-starved cells with IGF induces an increase in Akt phosphorylation in ΔCdh1 cells. Phospho-Akt (Ser473) and phospho-Akt (Ser308) were analysed on separate gels. For analysis of phospho-Akt (Ser473), blot lanes for additional CRISPR clones were removed, as denoted by the dashed line.

Enhanced PI3K/Akt signalling is a tumour-promoting event that is widely observed in several types of cancer. Activation of this pathway can be induced by either mutational activation or aberrant GFR-dependent signals. To study if Akt pathway activation could be induced by autocrine growth factor-dependent signals, we initially cultured our cell lines without serum and assayed phosphorylation of Akt (at residues Ser473 and Thr308). Interestingly, both mouse and human ILC cells displayed elevated Akt activation when compared to the E-cadherin-expressing cells (Fig. 3a). This trend was also observed for the downstream target mTOR, while expression of PTEN, a negative regulator of PI3K signalling, remained lower (Fig. 3a). To assess if mutational activation of core PI3K pathway components was underpinning pathway activation, we performed next-generation sequencing on mILC cell lines, which did not reveal somatic mutations in the GFR/PI3K/Akt pathway members *Igf1r*, *Pik3ca*, *Akt1*, *Akt2*, *Akt3*, *Pten* and *Mtor* (data not shown). In contrast, the human ILC cell line IPH-926 harboured a heterozygous deletion in *PTEN* (c.950_953delTACT) that probably contributes to the observed Akt activation under serum-free conditions. The high basal levels of phospho-Akt in human ILC cells, however, did not represent maximal activation, as administration of recombinant insulin-like growth factor (IGF) induced a further increase (Fig. 3b). These results indicate that loss of E-cadherin promotes constitutive Akt activation, even in the presence of PTEN-inactivating mutations.

Because we did not detect activating somatic mutations in PI3K pathway members in the mouse breast cancer cell lines, and E-cadherin loss induced an increase in phosphorylation of Akt under serum-free conditions, we hypothesised that an autocrine loop might underlie GFR-dependent Akt activation. To test this, we harvested conditioned medium from mILC cell lines that were cultured under serum-free conditions for 3 days. Similar experiments using the human ILC cells were not performed due to the extensive doubling time (approximately 14 days) and low metabolic activity *in vitro*^16^. Therefore, culture supernatant from serum-starved mILC cells was used to stimulate serum-starved Trp53^∆/∆^-3 cells, and Akt phosphorylation was assessed. Interestingly, mILC-conditioned medium induced a robust increase in phospho-Akt, while stimulation with Trp53^∆/∆^-conditioned medium did not affect Akt phosphorylation (Fig. 3c). These data thereby confirmed our assumption that GFR-dependent signalling is subject to autocrine activation upon mutational inactivation of E-cadherin. Subsequent mRNA expression profiling of the mouse and human ILC cell lines demonstrated that a number of GFR–ligand pairs were concomitantly expressed, such as transforming growth factor (TGF)-β receptors and TGFs, activin receptors and inhibins, and fibroblast growth factor (FGF) receptors and FGFs (Supplementary Table S4), which could underlie the autocrine activation observed. In summary, we conclude that autocrine GFR activation contributes to PI3K/Akt pathway activation in E-cadherin mutant ILC cells.

To establish causality and uncouple autocrine-induced growth factor-dependent signalling from oncogenic mutations, we undertook a CRISPR/Cas9-based knock-out strategy to ablate E-cadherin in mouse Trp53^∆/∆^ and human MCF7 cells (Supplementary Fig. S3). We assessed Akt phosphorylation upon stimulation with IGF because ILC cells respond well to this growth factor (Fig. 3b). Indeed, knock-out of E-cadherin (∆Cdh1) in the mouse Trp53^∆/∆^ cells increased Akt phosphorylation on Thr308 and Ser473 by 8.8- and 4.4-fold, respectively, upon stimulation with IGF (Fig. 3d). Knock-out of E-cadherin in the MCF7 cells also induced a higher (up to 2.0-fold) activation of Akt after IGF administration (Supplementary Fig. S3). However, because (in contrast to the mouse Trp53^∆/∆^ cells) MCF7 cells contain an activating *PIK3CA* mutation and *AKT1* amplification^17^, our data suggest that de-repression of GFR signalling upon E-cadherin loss has a modest effect on IGF-induced Akt activation in the presence of oncogenic GFR signalling. In short, our findings link loss of E-cadherin to hyperactivation of autocrine growth factor-dependent signals in ILC.

### IGF-1 expression is increased in human ILC versus IDC

**Figure 4:**
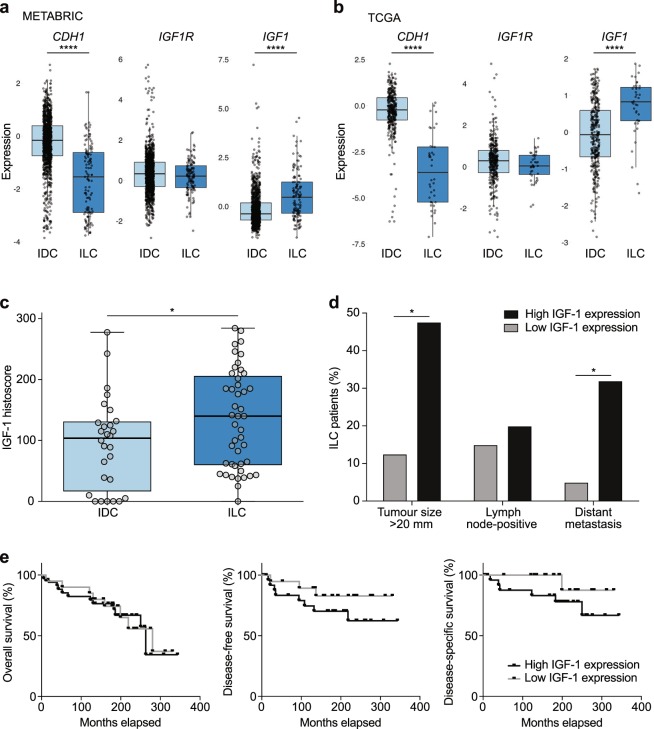
IGF-1 expression is increased in human ILC versus IDC. (**a**,**b**) Boxplots of expression for *CDH1*, *IGF1R* and *IGF1* genes from METABRIC (**a**) and TCGA (**b**) microarray mRNA expression datasets. All data points are ER-positive breast cancer samples. For further details, see Supplementary Table S5. Boxplots display the median (line), 25th and 75th percentiles (box) and 1.5 × interquartile range (whiskers). Light blue, IDC; dark blue, ILC. ^****^P < 0.0001; Wilcoxon test. (**c**,**d**) Analysis of IGF-1 cytoplasmic expression in a human TMA containing ILC and IDC samples. Boxplot (**c**) summarises IGF-1 histoscores. *P < 0.05; Mann–Whitney test. Histograms (**d**) show significant correlation between high cytoplasmic IGF-1 expression and increased tumour size and relapse with distant metastasis. ^*^P < 0.05; tumour size, Pearson’s chi-squared test; distant metastasis, Fisher’s exact test. Lymph node status, *P* = 0.633; Fisher’s exact test. For further details, see Supplementary Table S6. (**e**) Kaplan–Meier plots representing proportions of patient survival. Disease-free survival was defined as time from primary surgery to first occurrence of relapsed disease (loco-regional recurrence and/or distant metastasis), and disease-specific survival was defined as time from primary surgery to breast cancer-related death. Overall survival (left panel), *P* = 0.869; disease-free survival (middle panel), *P* = 0.210; disease-specific survival (right panel), *P* = 0.135; log-rank test.

Given the ability of IGF-1 to hyperactivate the PI3K/Akt pathway in E-cadherin mutant breast cancer cells, we analysed *IGF1* expression in the METABRIC^18^ and TCGA (http://cancergenome.nih.gov/) mRNA expression datasets (Fig. 4a,b, Supplementary Fig. S4 and Supplementary Table S5). Figure 4a,b represents microarray analyses of *CDH1*, *IGF1R* and *IGF1* mRNA expression in oestrogen receptor (ER)-positive ILC and IDC samples. As expected, *CDH1* was decreased in ILC compared to IDC (*P* < 0.0001). Analyses of the microarray datasets did not reveal a statistical difference in *IGFR1* expression between the groups. However, we saw an increase in *IGF1* expression in ILC relative to IDC in both the METABRIC and TCGA microarray datasets (*P* < 0.0001) (Fig. 4a,b and Supplementary Table S5). Moreover, when we analysed the TCGA RNA-Seq data, we found a similar increase in *IGF1* expression in ILC relative to IDC, and we observed a significant decrease in *IGF1R* expression in ILC relative to IDC (Supplementary Fig. S4 and Supplementary Table S5).

To further study IGF-1 expression in lobular carcinoma and its correlation with clinicopathological features, we generated a TMA containing 54 ILC and 52 IDC samples. Baseline characteristics of the patient cohort, rates of loco-regional recurrence and distant metastasis, sites of distant metastasis and survival of patients represented in the TMA are summarised in Table 3 and Supplementary Fig. S5. The TMA was stained for IGF-1, and ILC tumours exhibited significantly higher cytoplasmic expression of IGF-1 compared to IDC tumours (Fig. 4c); the median histoscore for IGF-1 was 140 (interquartile range, 59.7–205.8) in ILC and 103.8 (interquartile range, 16.5–131.0) in IDC (*P* = 0.0408). Patients were divided into those with high (above the median) or low (below or equal to the median) expression according to the overall median histoscores for IGF-1 expression. Pearson’s chi-squared analysis was then performed to determine whether increased IGF-1 expression correlated with clinicopathological variables (Supplementary Table S6). In all patients, increased cytoplasmic IGF-1 expression significantly associated with increased tumour size (*P* = 0.030). When ILC and IDC patients were analysed independently, this trend held for ILC (*P* = 0.024) but not for IDC. In ILC patients, high cytoplasmic IGF-1 expression also significantly correlated with relapse with distant metastasis (*P* = 0.030) (Fig. 4d and Supplementary Table S6). Finally, Kaplan–Meier analysis was undertaken to ascertain whether IGF-1 expression influenced survival outcomes. There was no association between IGF-1 expression and either disease-free survival or disease-specific survival (Fig. 4e). Thus, despite the positive association between cytoplasmic IGF-1 expression and the parameters of tumour size and distant metastasis in ILC patients, this did not impact upon survival outcomes.

**Table 3:** Clinicopathological features of the ILC and IDC patients studied for IGF-1 expression.

Clinicopathological characteristic^a^		All	ILC	IDC	*P*
Number of patients (*N*)	106	54	52	—	
Age (years)	Median	55.5	56.0	56.0	0.228
	≤50 (%)	26.4	31.5	21.2	
	>50 (%)	73.6	68.5	78.8	
Tumour size	≤2 cm (%)	71.7	67.4	75.5	0.391
	>2 cm (%)	28.3	32.6	24.5	
ER expression	Positive (%)	77.2	78.4	75.6	0.574
PR expression	Positive (%)	62.6	60.0	65.9	0.566
HER2 expression	Positive (%)	9.8	6.4	13.3	0.262
E-cadherin expression	Negative (%)	50.6	77.1	14.3	**0.000**
Lymph node status	Positive (%)	10.4	16.7	3.8	**0.030**
**Pattern of recurrence** ^**b**^		**All**	**ILC**	**IDC**	***P***
Local recurrence (*N*)		11	8	3	0.202
Regional recurrence (*N*)		3	2	1	1.000
Distant metastasis (*N*)		15	11	4	0.093
**Site of distant metastasis** ^**c**^		**All**	**ILC**	**IDC**	***P***
Lung		1	1	0	1.000
Bone		9	8^*^	1	**0.032**
Brain		1	1	0	1.000
Liver		3	1	2	0.064
Visceral		3	3^*^	0	0.243
Unknown site		1	0	1	—

^a^Comparison of the clinicopathological characteristics in ILC and IDC patients at baseline. *P*-values were calculated using Pearson’s chi-squared tests, except that for lymph node status, which was calculated using Fisher’s exact test. Protein expression proportions were calculated from scorable tumour cores. PR, progesterone receptor. ^b^Patterns of recurrence in ILC versus IDC patients. Local recurrence is defined here as relapsed disease within the same breast, and regional recurrence as axillary lymph node metastasis. *P*-values were calculated using Fisher’s exact tests. ^c^Sites of distant metastasis in ILC versus IDC. *P*-values were calculated using Fisher’s exact tests. Asterisks (*) indicate three patients that had both bone and visceral metastasis.

### ILC cells are sensitive to pharmacological inhibition of Akt

**Figure 5:**
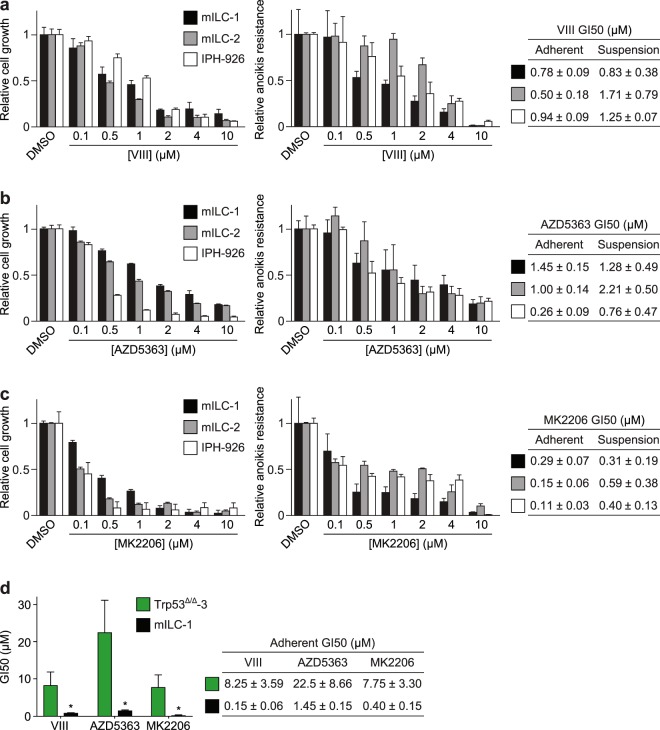
ILC tumour growth and survival is dependent on Akt activation. (**a**–**c**) Effect of Akt inhibitors VIII (**a**), AZD5363 (**b**) and MK2206 (**c**) on cell growth (left panels) and anoikis resistance (middle panels) of mILC-1 (black bars), mILC-2 (grey bars) and IPH-926 (white bars) cells. The GI50 values (µM) for each inhibitor in adherent and suspension settings are shown in tables (right panels). GI50 values were calculated based on three independent experiments. (**d**) E-cadherin inactivation induces sensitivity to pharmacological Akt inhibition in adherent cells. Shown is a comparison of GI50 values based on colony formation assays of Trp53^∆/∆^-3 cells (green bars) versus mILC-1 cells (black bars) (left panel). The GI50 values (µM) for each inhibitor for the cell types are shown in a table (right panel). Note the difference in sensitivity to the Akt inhibitors VIII, AZD5363 and MK2206. ^*^P < 0.05; Student’s *t*-test.

Given the increase in Akt activity, we wondered whether E-cadherin mutant breast cancer cells were sensitive to pharmacological inhibition of the PI3K/Akt pathway. To inhibit Akt, we used the ATP competitor AZD5363 and two allosteric inhibitors, MK2206 and VIII. The latter is being used *in vitro* to target Akt1/2 with high specificity, while AZD5363 and MK2006 are currently being tested in clinical trials^19,20^. We cultured the ILC cell lines in adherent and non-adherent (suspension) settings, which we employ as a surrogate readout for cell survival during metastatic dissemination^3^. Using VIII, AZD5363 or MK2206, we observed a dose-dependent inhibition of growth and survival in adherent and non-adherent conditions (Fig. 5a–c). Although all inhibitors induced a reduction in growth and survival, the strongest reduction in growth and survival was observed for MK2206, with a 50% growth inhibition (GI50) observed at concentrations below 500 nM in all cell types (Fig. 5c). In conjunction with previous results defining 0.5 µM to mark sensitivity to MK2206^21^, our results show that mouse and human ILC cells are among the most responsive anoikis-resistant breast cancer cell lines reported. Interestingly, when we compared adherent growing Trp53^∆/∆^ cells to mILC cells, we observed a clear difference in sensitivity to Akt-inhibiting drugs (Fig. 5d). Moreover, enhanced Akt responsiveness was also observed upon E-cadherin inactivation in Trp53^∆/∆^; ∆Cdh1 cells (Fig. 3d). As such, our results show that loss of E-cadherin causes a hypersensitive PI3K/Akt pathway, which sensitises cancer cells to pharmacological inhibition of Akt.

**Figure 6:**
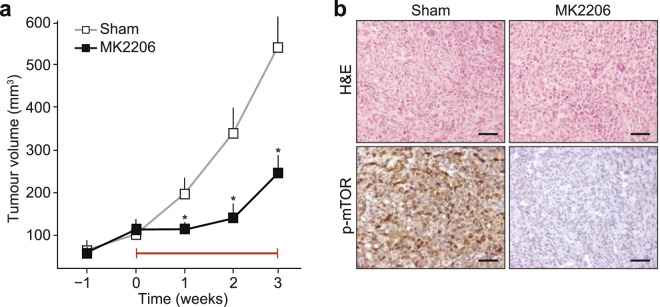
MK2206 inhibits tumour growth in a mouse model of ILC. (**a**) Mouse ILC cells (mILC-1) were allowed to form primary tumours in recipient nude mice, and treatment with MK2206 (120 mg/kg) was commenced when mammary tumours reached an average volume of 100 mm^3^ (*n* = 13). Sham-treated animals (*n* = 13) were used as control. Preclinical intervention was continued for three weeks (denoted by red line), after which the experiment was ended. ^*^P < 0.005. (**b**) Owing to the allosteric nature of the MK2206 inhibitor, inhibition of Akt signals in primary tumours was probed using immunohistochemical analysis of phosphorylated mTOR, a key downstream PI3K/Akt effector (lower panels). Note the inhibition of ILC growth (**a**) and phospho-mTOR (**b**) upon MK2206 treatment. H&E, haematoxylin and eosin staining. Sham, 30% captisol. Scale bars, 50 µm.

Finally, we performed a preclinical assessment of our findings by using the allosteric Akt inhibitor MK2206 in mice. To treat mILC, we orthotopically transplanted 20,000 mILC-1 cells and followed the mice until tumour volumes reached 100 mm^3^. Mice were then randomly assigned to a sham-treated group (*n* = 13) or a MK2206-treated cohort (*n* = 13). Inhibition of Akt using MK2206 led to a significant inhibition of tumour growth during the treatment (Fig. 6a) and local inhibition of phospho-mTOR, a key downstream effector of Akt signal (Fig. 6b).

In sum, our data demonstrate that E-cadherin loss is a driving cause of GFR hyperactivation in ILC, irrespective of oncogenic driver mutations. The resulting sensitivity to pharmacological Akt inhibition advocates the use of drugs targeting Akt as a treatment option for all ILC patients.

## Discussion

Current treatment for ILC is mostly directed against ERs, as ER is expressed in the majority of luminal tumours. Despite the expression of these favourable prognostic markers, the overall prognosis for ILC is comparable to other types of breast cancer owing to resistance to hormone receptor antagonists and low chemotherapeutic responsiveness^22,23^. However, prolonged parametric studies suggest that ILC has a worse survival than IDC when corrected for age, grade, TNM (tumour, node, metastasis) status and ER expression^24^. Although targeted therapy for breast cancer is widely studied, clinical success rates have remained low, which might be largely attributed to the lack of good biomarkers that enable adequate patient stratification. Because ILC is a major and specific breast cancer subset that is driven by loss of E-cadherin and has a distinct biomarker profile^25–29^, it is very well suited for targeted clinical intervention.

PI3K pathway inhibitors are and have been widely used as a targeted treatment option for breast cancer^30,31^. However, to our knowledge, this treatment option has neither been specifically probed for in ILC nor has it comprehensively been tested in ILC lacking GFR pathway mutations. Because we show that loss of E-cadherin directly contributes to the activation of growth factor-dependent Akt signalling – even to a modest extent in oncogene-addicted MCF7 cells – our data provide a clear potential for use of PI3K/Akt inhibitors in the complete ILC spectrum, irrespective of oncogenic mutations in the GFR/PI3K/Akt pathway.

Autocrine Akt phosphorylation upon E-cadherin inhibition can be caused by de-repression of GFR signals^32–34^, a feature that we have previously coupled to p120-catenin loss and subsequent AJ dismantling^35^. In addition, E-cadherin loss may promote reduced PTEN levels through junctional stability maintenance, resulting in increased Akt activation^36–38^. However, we have not obtained evidence for PTEN decrease as a direct result of E-cadherin knock-out. Our data indicate that Akt activation in ILC cells is instigated by an autocrine and growth factor-dependent PI3K/Akt cue. Interestingly, although GFR levels can be induced through Akt-dependent positive feedback signals^39,40^, our current and previously published data do not support this scenario in the context of AJ inactivation^35^.

Activation of PI3K signalling in cancer is often attributed to activating mutations, which are also frequently observed in ILC^7^. However, the fact that we mostly observed increased Akt phosphorylation upon loss of E-cadherin in the absence of PI3K/Akt pathway mutations implies that two complementary modes of activation may underlie PI3K/Akt activation in ILC. An opportunity to treat primary ILC and its disseminating cancer cells arises from the ability of MK2206 to potently restrain cell survival of ILC cell lines in both adherent and non-adherent settings. As a mono-therapy, MK2206 had a moderate effect on tumour growth in a breast xenograft model, while combination with paclitaxel further increased this anti-tumour effect^21,41^. In addition, phase I clinical trials combining MK2206 with paclitaxel, anastrozole or trastuzumab in advanced solid tumours and metastatic breast cancer reported anti-tumour activity with no serious adverse effects^19,20,42^. Although the response to MK2206 *in vitro* was highly increased in cell lines harbouring *PIK3CA* or *PTEN* mutations, tumour responses could not be linked to the presence of mutations in *PIK3CA*^21,43^. Also, the low number of ILC cases and the lack of central pathological validation of histotypes and E-cadherin status generally prevents firm conclusions regarding the efficacy of PI3K/Akt inhibition in ILC. An additional layer of complexity is added by the apparent lack of association between phospho-Akt levels in patients and mutational pathway activation^7,44,45^. These parameters further strengthen our assumption that AJ inactivation promotes PI3K/Akt activation and that hyperactivation through E-cadherin loss evokes differential dependency on PI3K/Akt signals when compared to cancers that are oncogene-addicted due to somatic mutations in GFR pathway effectors. Therefore, patient inclusion criteria in clinical trials targeting PI3K/Akt should be based on central pathology review for histological breast cancer subtypes. We propose to differentially diagnose ILC based on phenotype, AJ dysfunction and the activation of downstream effectors. Prime candidates to serve as biomarkers are established hallmarks, such as E-cadherin loss^46^ and cytosolic p120-catenin^47^, or markers that correspond to E-cadherin inactivation, such as nuclear localisation of phosphorylated cofilin^3^ or YAP^48^.

We identify the growth factor-dependent PI3K/Akt pathway as a prime target for the treatment of ILC. Because Akt activation is a direct consequence of E-cadherin loss, functional inactivation of E-cadherin and the AJ, rather than the presence of oncogenic mutations in this PI3K/Akt pathway, should be used as inclusion criteria for clinical PI3K/Akt intervention trials in this breast cancer subtype.

## Methods

### Cell culture

Mouse mammary carcinoma cells were cultured as described^14,15^. MCF7 (DSMZ no. ACC 115) and IPH-926 (DSMZ no. ACC 827) cells were obtained from DSMZ and were grown in DMEM-F12 (Sigma-Aldrich) containing 12% FCS (Sigma-Aldrich), supplemented with 100 IU/ml penicillin, 100 µg/ml streptomycin and 2.5 mM Ultraglutamine (Lonza). To generate E-cadherin knock-out cell lines, guide RNAs targeting human *CDH1* (GCTGAGGATGGTGTAAGCGATGG) and mouse *Cdh1* (CGTGTCATCAAATGGGGAAGCGG) were cloned into the pSicoR CRISPR/Cas9 vector^49^ using *BsmB*l restriction sites.

### Reverse-phase protein array analysis

Cells were washed with ice-cold phosphate-buffered saline (PBS) and lysed in 50 mM HEPES (pH 7.4), 1% Triton X-100, 10% glycerol, 150 mM sodium chloride, 1.5 mM magnesium chloride, 1 mM EGTA, 100 mM sodium fluoride, 10 mM sodium pyrophosphate, 1 mM sodium orthovanadate, supplemented with cOmplete ULTRA protease inhibitor and PhosSTOP phosphatase inhibitor cocktails (Roche), on ice. Lysates in biological triplicate were clarified by centrifugation (18,000 × *g*, 10 min, 4 °C), adjusted to 1 mg/ml concentration and serially diluted to produce a dilution series comprising four serial 2-fold dilutions of each sample. Sample dilution series were spotted onto nitrocellulose-coated slides (Grace Bio-Labs) in technical triplicate under conditions of constant 70% humidity using an Aushon 2470 arrayer (Aushon Biosystems). Slides were hydrated in blocking buffer (Thermo Fisher Scientific) and incubated with validated primary antibodies (Supplementary Table S1). Bound antibodies were detected by incubation with anti-IgG DyLight 800-conjugated secondary antibodies (New England BioLabs). Slides were read using an InnoScan 710-IR scanner (Innopsys), and images were acquired at the highest gain without saturation of the fluorescence signal. The relative fluorescence intensity of each sample spot was quantified using Mapix software (Innopsys). The linear fit of the dilution series of each sample was determined for each primary antibody, from which median relative fluorescence intensities were calculated for each technical replicate. Signal intensities were normalised across the panel of antibodies using global sample median correction^50^, and mean normalised intensities were calculated for each biological replicate.

### Western blotting

Protein samples were analysed by sodium dodecyl sulfate (SDS)–polyacrylamide gel electrophoresis and western blotting as previously described^51^. In addition to the antibodies used for RPPA analysis (Supplementary Table S1), the following antibodies were used for western blotting: rabbit anti-phospho-Akt (Ser473) (1:1,000; 5158, Cell Signaling Technology), goat anti-Akt (1:1,000; sc-1618, Santa Cruz Biotechnology) and mouse anti-GAPDH (1:10,000; mAb374, Millipore). Secondary antibodies were swine anti-rabbit-PO (p217, DAKO), goat anti-mouse-PO (170–6516, Bio-Rad), goat anti-rabbit-PO (170–6515, Bio-Rad) and rabbit anti-goat-PO (p160, DAKO). When total protein and phosphoprotein antibodies were used, samples were run on different blots using GAPDH as a normaliser, stripped of their primary antibodies using 62.5 mM Tris-HCl (pH 6.7), 2% SDS and 0.7% β-mercaptoethanol (10 min, 50 °C) or, when the primary antibodies were of two different species, treated with 1 mM NaN_3_ to inhibit peroxidase activity. See Supplementary Fig. S6 for original blots.

### Hierarchical cluster analysis

Mean normalised intensities were standardised by *Z*-score transformation. Normalised intensity values for phosphoproteins were further normalised to intensities of respective total proteins prior to standardisation. Unsupervised hierarchical cluster analysis of standardised protein abundances was performed using Euclidean distance and Cluster 3.0 (C Clustering Library, version 1.54)^52^, computing distances using an average-linkage matrix. Clustering results were visualised using Java TreeView (version 1.1.6)^53^.

### Conditioned medium stimulation assays

Cells were seeded in 6-well plates, serum-starved overnight and treated with conditioned medium or growth factors when cultures were approximately 80% confluent. Next, cells were lysed directly in lysis buffer containing 70 mM Tris-HCl (pH 7.4), 2% SDS, 4% glycerol and 0.5% β-mercaptoethanol. Cells were stimulated for 3 min with 100 ng/ml recombinant IGF (Gibco Life Technologies). For the collection of conditioned medium, cells were grown without serum for at least 48 h. Supernatant was harvested and cleared using a 45- µm filter.

### Immunohistochemistry

TMAs were collected and constructed as previously described^48,54^ and as follows. For the Utrecht TMA, haematoxylin and eosin-stained sections from primary ILC tumours were reviewed by a consultant breast pathologist and representative areas were identified. Construction of the TMA took place by extracting three individual tissue cores (0.6 mm) from these designated areas in the original paraffin-embedded tissue blocks, which were re-embedded into a recipient paraffin block. Approval for use of the TMA was granted by the Utrecht Tissue Governance Committee. The use of anonymous or coded left-over material for scientific purposes is part of the standard treatment contract with patients in The Netherlands, and no ethical approval is required according to Dutch legislation (as is stated by the Dutch committee for research on patient material ‘Centrale Commissie Mensgebonden Onderzoek’). For the Edinburgh TMA, approval for the construction of a TMA and its use for this analysis was granted by the NHS Lothian Tissue Governance Committee (SR184). The ethical approval for these tissues permits use of unconsented diagnostic archival tissue used in a de-identified manner. Suitable archival cases of 54 primary ILC and 52 IDC (both grade 2) were selected from the Edinburgh Breast Conservation Series – a fully documented, consecutive cohort of 1,812 patients treated by breast conservation surgery, axillary node sampling or clearance, and whole breast radiotherapy between 1981 and 1998. The majority of patients received adjuvant endocrine therapy, and 65.1% of patients were treated with tamoxifen. The remainder of patients received chemotherapy and endocrine therapy (8.4%), and 12.7% received no systemic treatment. A consultant breast histopathologist reviewed haematoxylin and eosin-stained sections of breast cancer specimens taken at the time of primary resection and identified representative areas. The TMA was constructed using individual tissue cores extracted from these designated areas in the original blocks and re-embedded into a recipient paraffin block. To take account of intra-tumour heterogeneity, triplicate cores were taken from two blocks per patient. For both TMAs, all methods were carried out in accordance with relevant guidelines and regulations from the two centres.

Immunohistochemical staining was performed as described previously^54^. Primary antibody incubation was done for 2 h at room temperature or overnight at 4 °C (rabbit anti-phospho-mTOR (Ser2448) (1:10; 49F9, Cell Signaling Technology), anti-IGF-1 (1:1,000; ab9572, Abcam) and rabbit anti-PTEN (1:100; 9559, Cell Signaling Technology) in 1% bovine serum albumin in PBS. HRP-conjugated secondary antibodies were Powervision poly-HRP anti-mouse, rabbit and rat (DPVO500-HRP, Immunologic) and poly-HRP anti-rabbit (DPVR500-HRP, Immunologic). These were incubated for 30 min at room temperature, followed by development using 3,3′-diaminobenzidine and haematoxylin for counterstaining. Immunohistochemistry scoring took place blinded to patient characteristics and previous staining results. Cytoplasmic IGF-1 expression was scored using the semi-quantitative weighted histoscore system. Intensity of the staining was assessed and graded as negative (0), weak (1), moderate (2) and strong (3). Tumour cell percentages within each category was then estimated and a histoscore calculated.

### Immunofluorescence

Cells were grown on glass cover-slips, washed with Ca^2+^- and Mg^2+^-containing PBS and fixed with 4% paraformaldehyde in PBS for 5 min at room temperature. Cells were permeabilized for 3 min using 0.3% Triton X-100 in PBS and blocked in 2% normal goat serum in PBS for 10 min. Blocking was performed by incubation in 10% normal goat serum in PBS for 10 min. Fixed samples were incubated overnight at 4 °C with primary antibodies in 1% bovine serum albumin in PBS using the following antibodies: mouse anti-p120-catenin (1:500; 610134, BD Biosciences) and Alexa Fluor 555-conjugated anti-E-cadherin (1:100; 560064, BD Biosciences). Goat anti-mouse-Alexa Fluor 488 (A11029, Invitrogen) was used as a secondary antibody and incubated for 1 h at room temperature. Samples were stained with DAPI for 3 min and mounted using Immu-Mount (Thermo Scientific). Samples were imaged using a Zeiss LSM 700 microscope (Carl Zeiss) and processed using ImageJ (National Institutes of Health) and Photoshop CS6 (Adobe).

### Pharmacological inhibitors

The following Akt inhibitors were used: AZD5363 (ITK Diagnostics), MK2206 (MedChemExpress) and VIII (Santa Cruz Biotechnology). All inhibitors were prepared as 10 mM stock solutions in DMSO. The final DMSO concentration did not exceed 0.1%.

### Anoikis assay

Anoikis assays were performed as previously described^15^. In short, cells were grown in suspension for 4 days, stained for apoptotic cells using Annexin V-FITC (IQ products) and propidium iodide and analysed by flow cytometry (PerkinElmer).

### Colony formation assay

Anchorage-dependent cell growth was assessed as previously described^5^. In short, cells were grown on 12-well plates incubated with the indicated inhibitors. When untreated cells reached confluence, the cells were fixed using methanol and stained with 0.2% crystal violet. ImageJ was used to quantify the surface area containing stained cells.

### Preclinical intervention studies

Recipient female nude mice (Hsd:Athymic Nude-Foxn1nu; Envigo) were anaesthetised with IsoFlo (isoflurane; Le Vet Pharma). The 4th (inguinal) mammary gland was exposed and approximately 10,000 mILC-1 cells^15^ were injected using a 10-μl Hamilton syringe. 0.1 mg/kg Temgesic (buprenorphine) was injected subcutaneous as analgesic treatment. Tumour development was measured using a digital calliper (Mitutoyo) on a weekly basis. When tumours reached a volume of 100 mm^3^, mice were sham-treated (30% captisol) or treated with MK2206 (120 mg/kg) three times per week on alternating days for 3 weeks. Mice were sacrificed when tumour volumes reached >500 mm^3^ or if mice presented detectable lung metastases using bioluminescence. All animal experiments were performed in accordance with local, national and European guidelines under permit AVD1150002015263 issued by The Netherlands Food and Consumer Product Safety Authority (NVWA) of the Ministry of Agriculture, Nature and Food.

### mRNA sequencing

Cells were seeded on a 6-well plate and grown to 80% confluence in serum-containing medium. After washing in Ca^2+^- and Mg^2+^-containing PBS, RNA was isolated and purified using the RNAeasy kit (Qiagen), followed by DNase treatment (Qiagen). After measurement of RNA concentration using a Qubit fluorometer (Invitrogen), 250 ng of total RNA was treated using a Ribo-Zero rRNA removal kit (Epicenter) to remove ribosomal RNAs. Sixteen microlitres of purified RNA was fragmented by addition of 4 μl 5× fragmentation buffer (200 mM Tris acetate (pH 8.2), 500 mM potassium acetate and 150 mM magnesium acetate) and incubated at 94 °C for exactly 90 s. After ethanol precipitation, fragmented RNA was mixed with 5 μg random hexamers, followed by incubation at 70 °C for 10 min and chilling on ice. From this RNA primer mix, first-strand cDNA was synthesised by adding 4 μl 5× first-strand buffer, 2 μl 100 mM DTT, 1 μl 10 mM dNTPs, 132 ng actinomycin D and 200 U SuperScript III in a Qiagen MinElute column to remove dNTPs and eluted in 34 μl elution buffer. Second-strand cDNA was synthesised by adding 91.8 μl H_2_O, 5 μg random hexamers, 4 μl 5× first-strand buffer, 2 μl 100 mM DTT, 4 μl 10 mM dNTPs with dTTP replaced by dUTP, 30 μl 5× second-strand buffer, 40 U *E. coli* DNA polymerase, 10 U *E*. *coli* DNA ligase and 2 U *E*. *coli* RNase H, and incubated at 16 °C for 2 h, followed by incubation with 10 U T4 polymerase at 16 °C for 10 min. Double-stranded cDNA was purified using a Qiagen MinElute column and used for Illumina sample preparation and sequencing according to the Illumina protocol. Before the final PCR, a band corresponding to ~300 bp (DNA + adaptor) was collected and incubated with 1 U USER enzyme (NEB) at 37 °C for 15 min, followed by 5 min at 95 °C. The 300-bp libraries were used for cluster generation on a HiSeq 2000 (Illumina). RNA-Seq reads were uniquely mapped to the human (hg19) and mouse (mm9) reference genomes using the Eland or BWA program, allowing 1 mismatch, and subsequently used for bioinformatic analysis. RPKM (reads per kilobase of gene length per million reads) values^55^ for RefSeq genes were computed using tag counting scripts and used to analyse the expression level of genes.

### Mutation analysis

Genomic DNA was isolated by standard proteinase K digestion and column purification (QIAamp DNA mini kit). Control DNA was obtained from a female *Wcre;Cdh1*^*F/F*^*;Trp53*^*F/F*^ mouse liver^14^. Gene panels were designed using the Ion AmpliSeq Designer website (v4.2 Ampliseq.com) and mapped to the human (hg19) and mouse (mm10) reference genomes. Amplicon libraries were synthesised using standard Ampliseq protocols (Thermo Fisher). In short, amplicons were amplified, followed by digestion of the primers using FuPa reagents (Thermo Fisher). Sequence adaptors and barcodes were ligated to the digested amplicons, and a short amplification and size selection was performed using AMPure beads (Beckman Coulter) to select complete and specific libraries. All libraries were pooled equimolarly and used for emulsion PCR on a OneTouch 2 system using the Ion PGM template OT2 400 kit (Thermo Fisher). After loading the live Ion Sphere Particles on an Ion 318 chip, sequencing was performed on an Ion Torrent PGM using Ion PGM 400 sequencing chemistry (Thermo Fisher), after which standard coverage analysis and variant-caller settings were used to detect variations. After variant calling, functional consequences were analysed using the Variant Effect Predictor (Ensembl). For mouse variants predicted to have a functional consequence, the corresponding human sequence was analysed for the presence of known somatic mutations in cancer (COSMIC).

### mRNA expression analysis

METABRIC (Illumina v3 microarray) and TCGA provisional (microarray and RNA-Seq v2) mRNA expression data (expression *Z*-scores; *Z*-score threshold ±2) were extracted from the cBioportal website (http://www.cbioportal.org/) for the genes of interest. Analysis was reduced to IDC and ILC ER-positive samples only. Boxplots were generated using the ggplot2 package in R.

### Statistical analyses

Differences between protein relative abundances derived from RPPA data were assessed by Student’s *t*-tests using the two-step linear step-up procedure of Benjamini, Krieger and Yekutieli to set the false discovery rate (FDR) at 5%. Differences between categories in TMAs were assessed by Pearson’s chi-squared tests or Fisher’s exact tests. Differences between gene expression derived from microarray and RNA-Seq data were assessed by Wilcoxon tests. For Kaplan–Meier analyses, log-rank tests were performed. Differences between histoscores were assessed by Mann–Whitney tests. For the calculation of GI50 values (50% growth inhibition or 50% anoikis), Prism 5 (GraphPad) was used. For statistical analysis of inhibitor assays, Student’s *t*-tests were performed. *P* < 0.05 was considered significant.

## Electronic supplementary material

Supplementary information

Supplementary Table S1

Supplementary Table S2

Supplementary Table S3

Supplementary Table S4
