# Supplementary material for: E-cadherin loss induces targetable autocrine activation of growth factor signalling in lobular breast cancer
Source: Sci Rep. 2018 Oct 18;8:15454. doi: 10.1038/s41598-018-33525-5 (PMC6193986; doi:10.1038/s41598-018-33525-5)
Supplement: Supplementary file 1 — Supplementary information [file 41598_2018_33525_MOESM1_ESM.pdf]

E-cadherin loss induces targetable autocrine activation of growth factor signalling in lobular breast cancer

Katy Teo[1,*], Laura Gómez-Cuadrado[1,*], Milou Tenhagen[2,*], Adam Byron[1,*], Max Rätze[2], Miranda van Amersfoort[2], Jojanneke Renes[2], Eric Strengman[2], Amit Mandoli[3], Abhishek A. Singh[3], Joost H. Martens[3], Hendrik G. Stunnenberg[3], Paul J. van Diest[2], Valerie G. Brunton[1,#] & Patrick W. B. Derksen[2,#]

[1]Cancer Research UK Edinburgh Centre, Institute of Genetics and Molecular Medicine, University of Edinburgh, Edinburgh, United Kingdom. [2]Department of Pathology, University Medical Center Utrecht, Utrecht, The Netherlands. [3]Department of Molecular Biology, Faculty of Science, Nijmegen Centre for Molecular Life Sciences, Radboud University, Nijmegen, The Netherlands. *Katy Teo, Laura Gómez-Cuadrado, Milou Tenhagen and Adam Byron contributed equally to this work. [#]Correspondence and requests for materials should be addressed to V.G.B. (email: v.brunton@ed.ac.uk) or P.W.B.D. (email: pderksen@umcutrecht.nl)

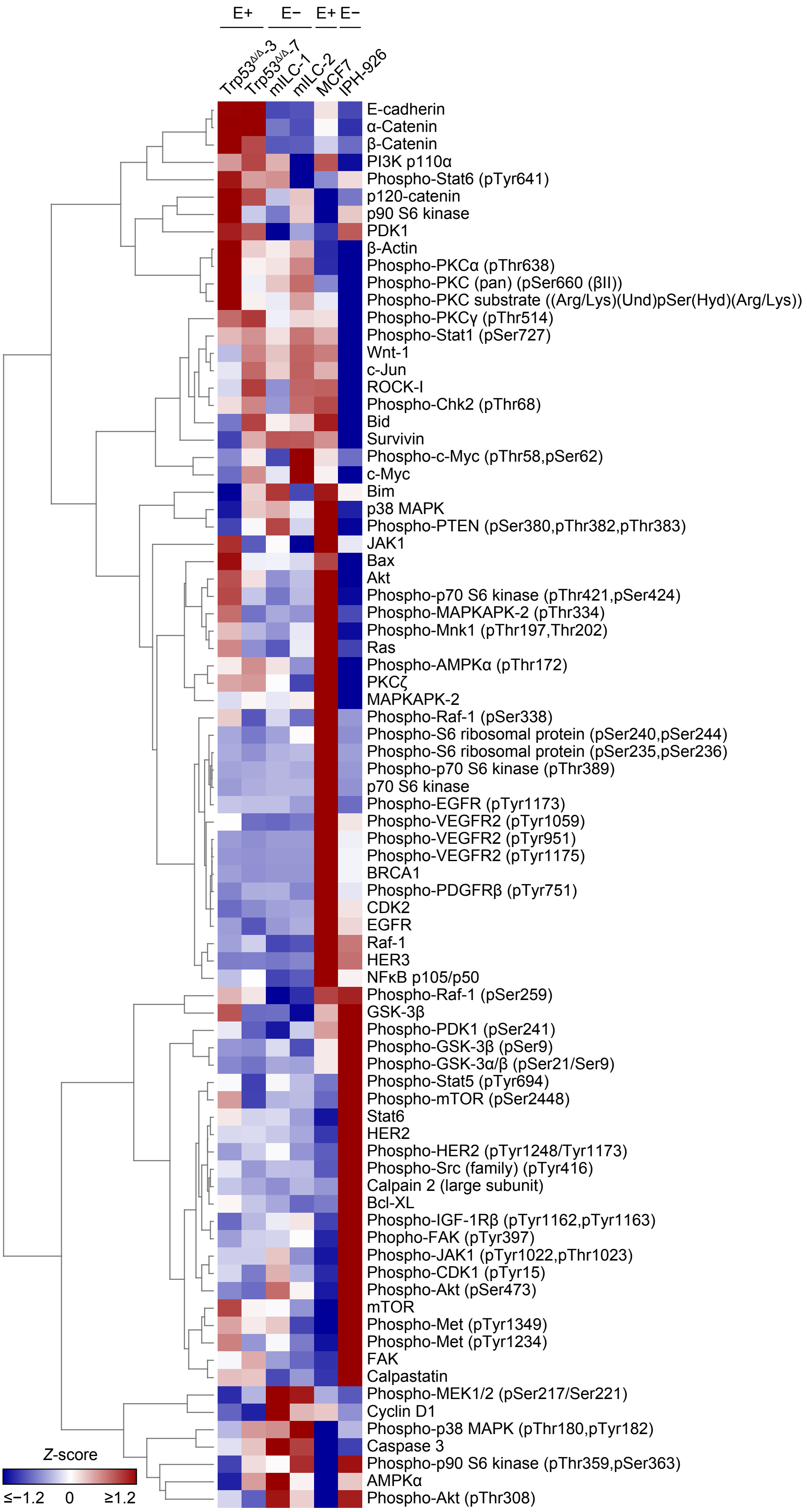

Supplementary Figure S1. Relative protein expression and phosphorylation in the context of E-cadherin expression. Heat map of RPPA data of phosphorylated and total protein expression comparing E-cadherin-positive (E+) and E-cadherin-negative (E−) human and mouse breast cancer cell lines. Normalised intensity values were standardised as *Z*-scores, and proteins/phosphoproteins were subjected to hierarchical cluster analysis. Heat maps display the relative expression of proteins or phosphoproteins (red, up-regulated; blue, down-regulated).

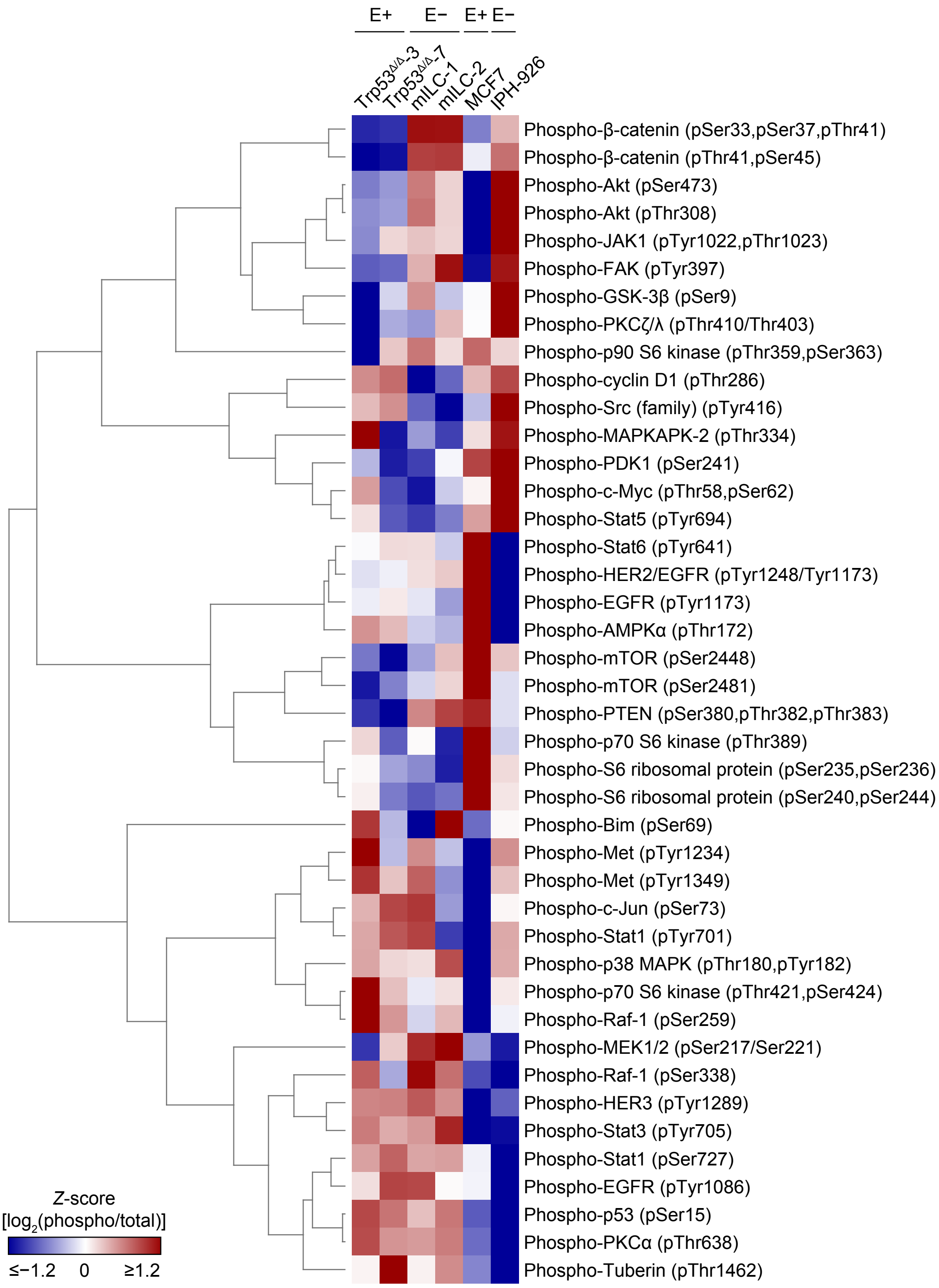

Supplementary Figure S2. Normalised relative phosphoprotein levels upon loss of E-cadherin expression. Levels of phosphoproteins relative to respective total proteins levels in whole cell lysates from E-cadherin-positive (E+) and E-cadherin-negative (E−) cells were determined by RPPA. Normalised intensity values for phosphoproteins were further normalised to intensities of respective total proteins as log_2-transformed ratios (phosphoprotein/total protein) and standardised as *Z*-scores, and phosphoproteins were subjected to hierarchical cluster analysis. Heat maps display the relative expression of phosphoproteins (red, up-regulated; blue, down-regulated).

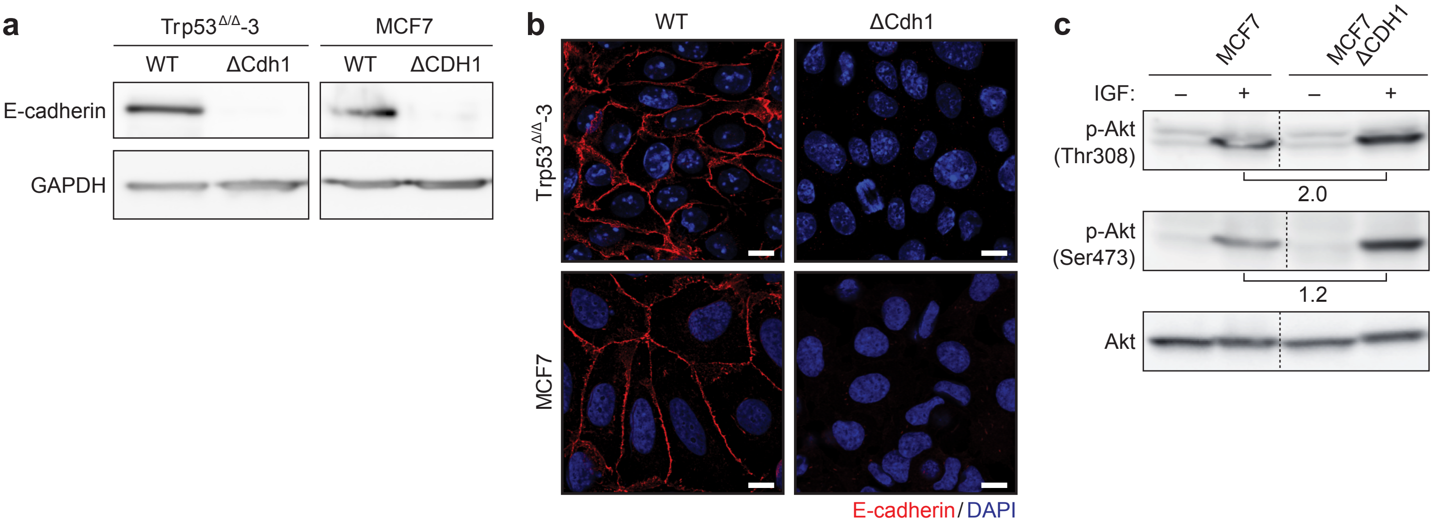

Supplementary Figure S3. CRISPR/Cas9 targeting of the E-cadherin locus in human and mouse breast cancer cells. (**a**) Expression of E-cadherin assessed by western blotting in Trp53$^{\Delta/\Delta}$-3 and MCF7 cells upon E-cadherin knock-out (ΔCDH1). GAPDH was used as loading control. (**b**) Immunofluorescence analysis showing E-cadherin expression (red) in E-cadherin knock-out and wild-type Trp53$^{\Delta/\Delta}$-3 and MCF7 cells. Scale bar, 10 µm. (**c**) E-cadherin knockout (ΔCDH1) in MCF7 cells, and stimulation of serum-starved cells with IGF. Phosphorylation of Akt (Thr308 and Ser473) was normalised over total protein levels.

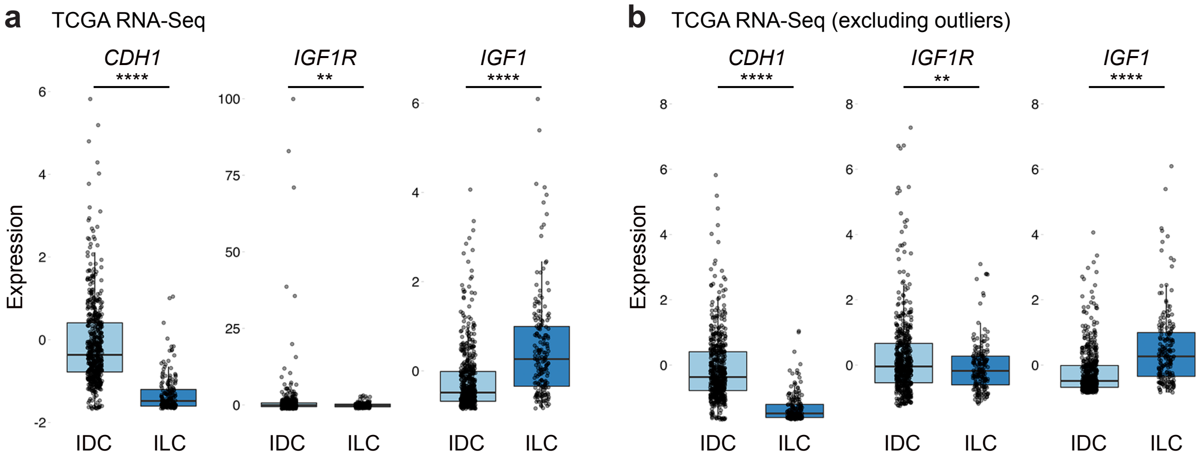

Supplementary Figure S4. Analysis of RNA-Seq data. (**a**) Boxplots of expression for *CDH1*, *IGF1R* and *IGF1* genes from TCGA RNA-Seq mRNA expression dataset. (**b**) Analysis of the same data as for **a**, excluding outlier samples from *IGF1R* gene expression. All data points are ER-positive breast cancer samples. For further details, see Supplementary Table S5. Boxplots display the median (line), 25th and 75th percentiles (box) and 1.5 × interquartile range (whiskers). Light blue, IDC; dark blue, ILC. $^{**}P < 0.01$, $^{****}P < 0.0001$; Wilcoxon test.

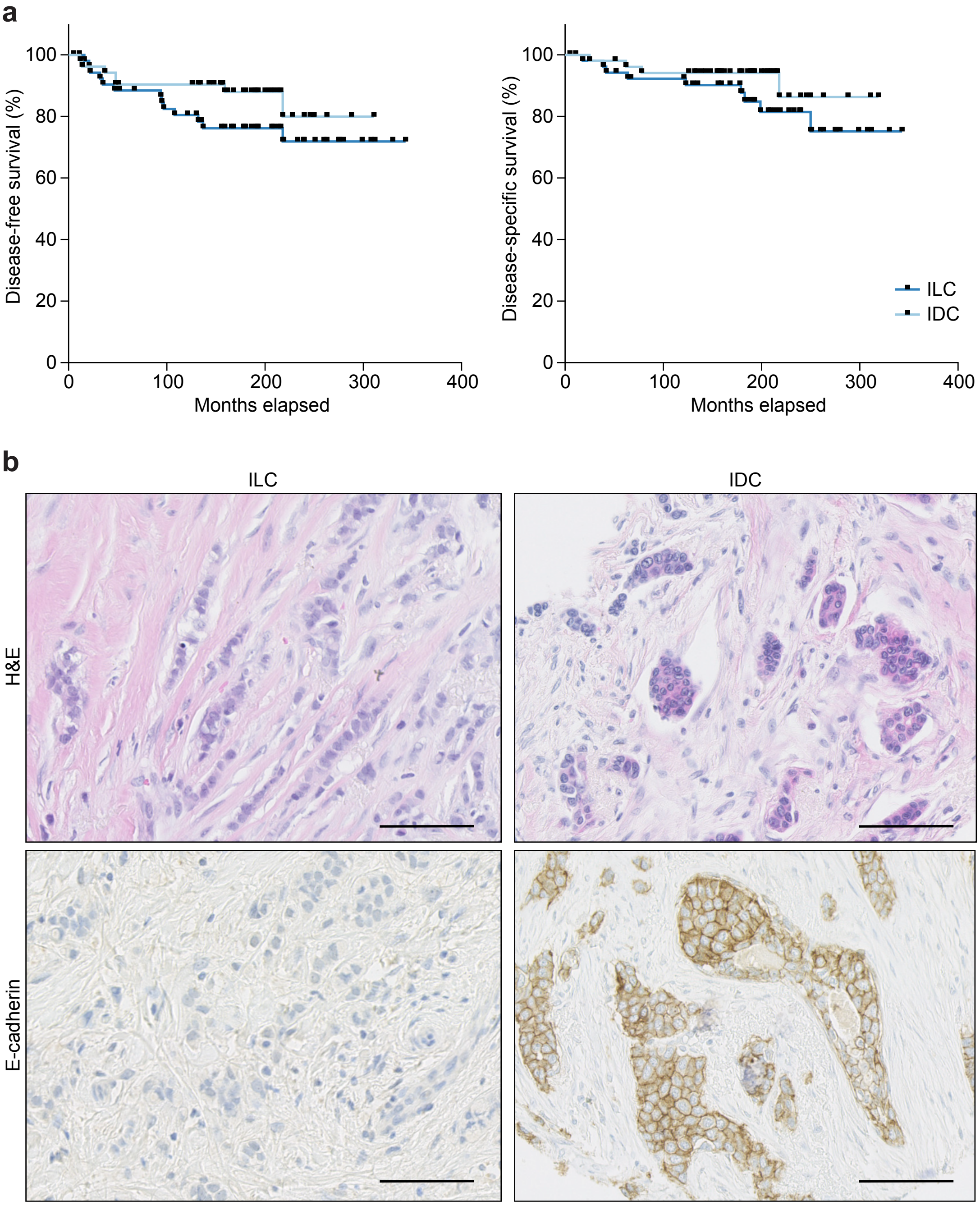

Supplementary Figure S5. Analyses of the ILC and IDC patients and TMA samples studied for IGF-1 expression. (**a**) Kaplan–Meier plots representing proportions of patient survival, stratified by histological subtype (ILC or IDC). Disease-free survival was defined as time from primary surgery to first occurrence of relapsed disease (loco-regional recurrence and/or distant metastasis), and disease-specific survival was defined as time from primary surgery to breast cancer-related death. Disease-free survival (left panel), $P = 0.177$; disease-specific survival (right panel), $P = 0.227$; log-rank test. (**b**) Representative immunohistochemistry images of ILC (left panels) and IDC (right panels) patient tumour cores showing loss of membranous E-cadherin expression in ILC. H&E, haematoxylin and eosin staining. Scale bars, 100 μm. For further details, see Table 3.

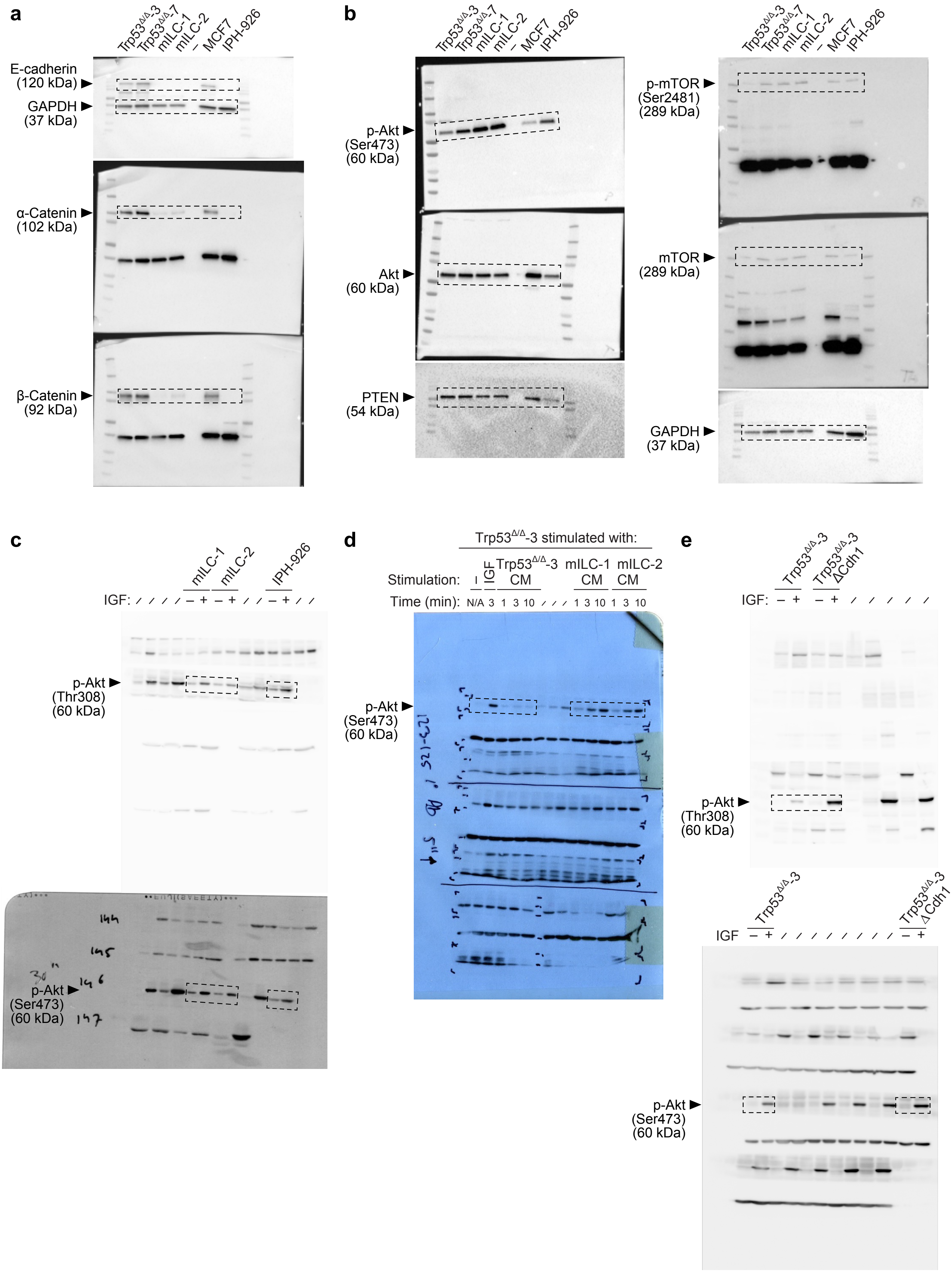

Supplementary Figure S6. Original blots. (**a–e**) Dashed boxes indicate cropped regions shown in Fig. 1b (**a**), Fig. 3a (**b**), Fig. 3b (**c**), Fig. 3c (**d**) and Fig. 3d (**e**). Arrowheads indicate expected band position for each protein probed. N/A, not applicable.

mRNA expression data source	Number of samples			*P* (IDC vs ILC)[a]		
	Total	IDC ER-positive	ILC ER-positive	***CDH1***	***IGF1R***	***IGF1***
METABRIC (Illumina microarray)	2,509	1,116	125	6.41×10^{-28}	2.12×10^{-1}	3.44×10^{-11}
TCGA (microarray)	529	340	40	1.17×10^{-17}	8.00×10^{-2}	4.49×10^{-7}
TCGA (RNA-Seq)	1,100	558	190	1.01×10^{-63}	9.45×10^{-3}	3.10×10^{-23}

Supplementary Table S5. mRNA expression data statistics. [a]Differences between gene expression were assessed by Wilcoxon tests.

Feature	P [a]		
	**All**	**ILC**	**IDC**
Age	0.193	0.419	0.305
Tumour size	**0.030**	**0.024**	0.484
ER expression	0.845	0.703	0.508
PR expression[b]	0.728	0.353	0.102
HER2 expression	0.781	0.409	0.520
E-cadherin expression	0.445	0.970	0.617
Lymph node status	0.383	0.633	1
Distant metastasis	0.104	**0.030**	0.524
p-Akt	0.077	0.097	1

Supplementary Table S6. TMA analysis of cytoplasmic IGF-1 expression. [a]Differences between categories were assessed by Pearson's chi-squared tests, except for lymph node status and distant metastasis, for which Fisher's exact tests were used. [b]PR, progesterone receptor.
